# Pharmacogenomics of 3,4-Methylenedioxymethamphetamine (MDMA): A Narrative Review of the Literature

**DOI:** 10.3390/pharmaceutics16081091

**Published:** 2024-08-20

**Authors:** Guillaume Drevin, Maria Pena-Martin, Aurélien Bauduin, Antoine Baudriller, Marie Briet, Chadi Abbara

**Affiliations:** 1 Service de Pharmacologie-Toxicologie et Pharmacovigilance, Centre Hospitalo-Universitaire d’Angers, 49100 Angers, France; mariacelsa@usal.es (M.P.-M.); aurelien.bauduin@chu-angers.fr (A.B.); antoine.baudriller@chu-angers.fr (A.B.); marie.briet@chu-angers.fr (M.B.); chadi.abbara@chu-angers.fr (C.A.); 2 Faculté de santé, Département médecine, Université d’Angers, 49100 Angers, France; 3 UMR INSERM 1083, CNRS 6015, Laboratoire MitoVasc, 49100 Angers, France

**Keywords:** 3,4-methylenedioxymethamphetamine, MDMA, pharmacogenomics, pharmacokinetics, pharmacodynamics

## Abstract

3,4-Methylenedioxymethamphetamine (MDMA) is a synthetic amphetamine derivative with notable psychoactive properties and emerging therapeutic potential, particularly for treating post-traumatic stress disorders (PTSD) and substance use disorders. However, its use remains controversial due to inter-individual variability influenced by both environmental and genetic factors. In this context, pharmacogenomics could play a crucial role in guiding MDMA treatment by identifying individuals with genetic predispositions affecting their response to MDMA. Tailoring treatment plans based on individual’s genetic makeup may enhance therapeutic outcomes and minimize adverse effects, leading to safer and more effective use of MDMA in clinical settings. Literature analysis reveals that the influence of genetic variants within genes encoded for enzymes involved in MDMA metabolism and/or pharmacodynamics (PD) targets have been relatively under-investigated in humans. Some studies have pointed out associations between MDMA-induced effects and polymorphisms. For example, the catechol-O-methyltransferase (COMT) Val158Met polymorphism has been associated with cognitive and cardiovascular MDMA-induced effects. Similarly, polymorphisms in the serotonin-linked promoter region (5HTTLPR) have been associated with several MDMA-induced adverse effects including mood disorders. However, despite these findings, only a few associations have been highlighted. Furthermore, some genes encoded for MDMA targets have been only poorly investigated, representing a significant research gap. These observations underscore the need for large-scale, controlled pharmacogenomics studies focusing on a broad panel of genes involved into MDMA pharmacokinetics and PD. Such studies could provide critical insights for optimizing MDMA’s therapeutic use and minimizing its risks.

## 1. Introduction

**Figure 1:**
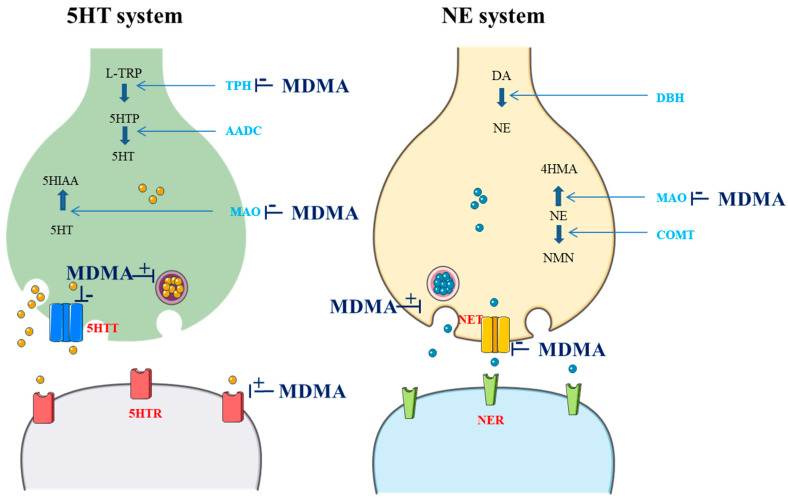
Effects of MDMA on the 5HT and NE system (drawn in part using images from Servier Medical Art. Servier Medical Art by Servier is licensed under a Creative Commons Attribution 3.0 Unported License (https://creativecommons.org/licenses/by/3.0/; accessed 8 August 2024)*;* L-TRP: L-tryptophan; TPH: L-tryptophan hydroxylase; 5HTP: 5-hydroxytryptophan; AADC: L-aromatic amino acid decarboxylase; 5HT: serotonin; MAO: monoamine oxidase; 5HIAA: 5-hydroxyindoleacetic acid; 5HTT: 5HT transporter; 5HTR: 5HT receptor; DA: dopamine; DBH: dopamine beta-hydroxylase; COMT: catechol; NE: norepinephrine; 4HMA: 4-hydroxymandelic acid; NMN: normetanephrine; NET: NE transporter; NER: NE receptor).

3,4-Methylenedioxymethamphetamine (MDMA) is a ring-substituted amphetamine first synthesized in 1912 by the German pharmaceutical company Merck as an intermediate to the styptic compound methylhydrastitine [1]. MDMA acts as a releaser and reuptake inhibitor of serotonin (5HT), norepinephrine (NE), and, to a lesser extent, dopamine (DA). MDMA can also inhibit both monoamine oxidase (MAO) and L-tryptophan hydroxylase (TPH). Furthermore, it seems to have direct actions on several receptors, including the serotonin 1A (5HTR1A), 1B (5HTR1B), and 2A receptors (5HTR2A), the M1 muscarinic receptor, the H1 histamine receptor, the α- and β-adrenergic receptors, and DA receptors (DR) [2,3,4,5]. Actions on cholinergic, glutamatergic, and gamma-aminobutyric acid receptors have also been reported [4]. Additionally, MDMA stimulates the release of oxytocin from the hypothalamus, increasing its level in the brain and bloodstream. This release is believed to significantly contribute to the enhanced feelings of social connection and empathy that users experience [6]. Overall, typical effects of MDMA can be predominantly attributed to the activation of the 5HT and NE systems [7]. These effects are summarized in the Figure 1.

**Figure 2:**
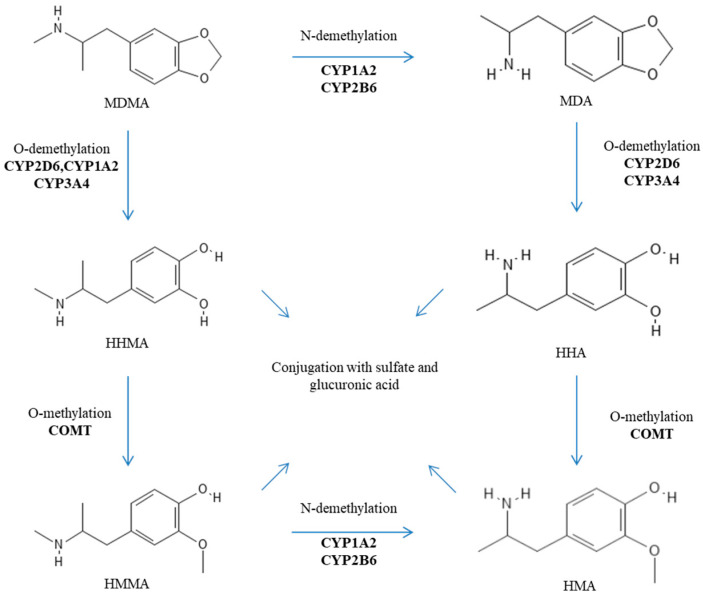
Metabolic pathways of MDMA.

After oral administration, MDMA is rapidly absorbed into the bloodstream (with a Tmax of about 2 h), and its transport is not mediated by P-glycoprotein (also known as MDR1 or ABCB1) [8,9]. MDMA is characterized by nonlinear pharmacokinetics (PK). This nonlinear PK is observed within the normal dosing range and becomes more pronounced at higher, and potentially toxic, doses. Indeed, De la Torre et al. reported nondose-proportional area under the curve (AUC) values for oral doses of 50, 75, 100, 125, and 150 mg, which were 9.15, 17.8, 18.5, 21, and 34.5, respectively [10]. Furthermore, its metabolism is rather complex and includes two main metabolic pathways: (i) O-demethylation to 3,4-dihydroxymethamphetamine (HHMA) followed by conversion to 4-hydroxy-3-methoxyamphetamine (HMA) after several additional steps; and (ii) N-demethylation to 3,4-methylenedioxyamphetamine (MDA) before ending up as HMA [2,3]. MDMA metabolites are eventually excreted in urine as conjugated glucuronide and/or sulfate metabolites [2,3]. These processes appear rather stereoselective with a preference for S-stereoisomers and depend on several enzymes including catechol-O-methyltransferase (COMT), cytochromes P450 (CYPs) (especially the isoforms 2D6, 2B6, 2C19, 3A4, and 1A2), glutathione S-transferase (SULT), and diphospho (UDP)-glucuronosyltransferase (UGT) [4,11,12,13]. The metabolic pathways of MDMA are reported in Figure 2. It is important to note that MDA, HHMA, and HMA are pharmacologically active metabolites [4,11,12,13]. In addition, HHMA (and to a minor extent HHA) can undergo further oxidation to orthoquinones, which, when conjugated to glutathione, display cytotoxicity and contribute to cell apoptosis [4,14,15]. Detoxification of such oxidized metabolites is dependent on glutathione S-transferases (GSTs) [13].

Acting as a stimulant and hallucinogen drug, MDMA has been pharmacologically characterized as an entactogen by the chemists Alexander Shulgin and David E. Nichols in the 1970s [16,17]. Since, it has been used actively as a psychotherapy tool by the psychedelic community until the early 1980s. However, due to its diversion towards a recreational drug and its abuse in the club scene as “Ecstasy”; the Drug Enforcement Administration (DEA) declared an emergency ban on MDMA in 1985 which, subsequently, became permanent thereafter, placing this compound on the list of Schedule I drugs (defined as substances with no currently accepted medical use and a high potential for abuse) [17]. Nowadays, the therapeutic use of MDMA remains rare or almost nonexistent worldwide [18]. However, MDMA is regaining a certain interest among psychiatrists, and several clinical trials are ongoing to explore whether MDMA has a therapeutic potential, especially in the treatment of post-traumatic stress disorder (PTSD), alcohol use disorder, and anxiety [19]. The data from a recent randomized placebo-controlled phase 3 trial suggest that MDMA-assisted therapy reduced PTSD symptoms and functional impairment in a diverse population with moderate to severe PTSD [20]. Moreover, MDMA assisted therapy could be of potential interest in cases of adult addictions like alcohol use disorder [19,21]. Such a therapeutic use of MDMA raises safety concerns. Indeed, the response to this substance seems to vary greatly among individuals and may be associated with transient neurocognitive effects including verbal and spatial memory deficits, slow processing speeds, and executive functioning impairments [19]. Furthermore, related fatalities have been reported with the illicit used of MDMA [22,23]. Such inter-variability seems to be influenced by both environmental and genetic factors. Environmental factors may include concomitant substance use or individual health status, while genetic factors primarily involve variations within genes encoding for MDMA’s metabolic enzymes and/or pharmacological targets [24,25]. In this context, pharmacogenomics could play a crucial role in guiding MDMA treatment by identifying individuals with genetic predispositions that may affect their response to MDMA. By tailoring treatment plans based on individual’s genetic makeup, it may be possible to enhance therapeutic outcomes and minimize adverse effects, leading to safer and more effective use of MDMA in clinical settings.

The objective of this narrative review is to identify and synthesize existing literature evaluating the pharmacogenomics of MDMA response. Identifying key allelic variants that may impact the PK and pharmacodynamics (PD) of MDMA could help to predict individual response and adapt the treatment, aiming to minimize adverse effects and maximize therapeutic benefits.

## 2. Genetic Factors Influencing the Metabolism of MDMA

Over the last decades, a certain interest has been focused on the pharmacogenomics of MDMA. Current research predominantly explores genetic factors impacting MDMA metabolism, notably polymorphisms within the genes encoding for COMT and CYP2D6. Tucker et al. first proposed that carrying the poor CYP2D6 metabolizer genotype could predispose individuals to acute MDMA toxicity and related death [26]. Such a postulate seems plausible given the highly polymorphic nature of the CYP2D6 gene, with more than 170 variants described (pharmVAR.org). These variants include single-nucleotide polymorphisms, small insertions/deletions, as well as larger structural variants such as multiplications, deletions, tandem arrangements, and copy number variations (CNV). The frequency of these variants differs across populations, and they significantly influence the drug-metabolizing function of CYP2D6 [27,28,29]. However, this hypothesis was not confirmed by further studies [30,31,32]. Indeed, O’Donohoe et al. aimed to determine whether individuals with poor metabolizer genotypes for the CYP2D6 enzyme were at higher risk of MDMA toxicity [31]. Thus, they retrospectively examined seven cases of toxicity or death thought to be due to MDMA. DNA was extracted from blood samples, amplified using polymerase chain reaction, and analyzed for the presence of CYP2D6 mutations “A” and/or “B”. In this study, none of the seven cases exhibited poor metabolizer genotypes for CYP2D6. According to the authors, such results suggested that CYP2D6 genetic variability did not significantly influence MDMA toxicity [31]. Although the specific CYP2D6 mutations referred to as “A” and “B” were not clearly identified in the study, they likely correspond to the CYP2D6*3 and CYP2D6*4 alleles based on the described genotyping method. Indeed, a polymerase chain reaction (PCR) technique followed by enzyme digestion was performed to detect these specific mutations. Bands at 280 bp and 160 bp indicate the A mutation (CYP2D6*3) while bands at 380 bp and 180 bp indicate the B mutation (CYP2D6*4). These alleles are associated with poor metabolizer phenotype. Nevertheless, the small sample size of only seven cases strongly limits the reliability and generalizability of the conclusions [31]. Further research with a larger cohort is necessary to validate these findings. Similarly, Gilhooly et al. also investigated if CYP2D6 enzyme deficiency could contribute to ecstasy-related fatalities [32]. They selected fifteen individuals believed to have died from ecstasy-related toxicity (defined by the pathologist as the presence of MDMA, MDA, and/or 3,4-methylenedioxy-N-ethylamphetamine). Biological materials (liver or blood) were genetically screened for the two nonfunctional CYP2D6*3 and CYP2D6*4. It is important to note that the study refers to alleles CYP2D6*3 and CYP2D6*4 without specifying the exact rsID numbers. In this study, none of the 15 genotyped samples were predicted to be a poor metabolizer. According to the authors, this result suggested that CYP2D6 deficiency did not appear to be a determinant factor in ecstasy-related fatalities [32]. However, here again, this study has notable limitations. The small sample size of only 15 subjects limits the ability to draw generalizable conclusions. Furthermore, the study focused solely on the CYP2D6*3 and CYP2D6*4 polymorphisms, neglecting other potentially relevant genetic variations that might influence MDMA metabolism and toxicity. Additionally, the complexity of MDMA-related fatalities, which can involve various mechanisms and are often associated with concurrent polydrug use, further limits the extent and applicability of the findings [32].

To date, the real impact of CYP2D6 genetic polymorphisms in explaining inter-individual differences in response to MDMA remains unclear. While some genetic polymorphisms have been shown to influence MDMA metabolism in humans, resulting in altered plasma concentrations of MDMA and/or metabolites (especially MDA and HHMA), their clinical relevance remains a matter of debate [4,33,34,35,36,37,38,39,40,41,42,43,44]. Indeed, some studies have downplayed the potential contribution of CYP2D6 polymorphisms in this response [40,41]. However, others have emphasized their significance [42,43,44]. For instance, low-activity CYP2D6 genotypes have been associated with an increased risk of hyponatremia and increased cortisol production when MDMA is used [42,44]. It is important to note that both studies do not specify rsID numbers for the CYP2D6 polymorphisms but group them into metabolizer status categories (poor, intermediate, extensive, and ultrarapid metabolizers). Conversely, in another study conducted by Cuyas et al. among a cohort of 263 subjects including 60 MDMA users in order to clarify the potential role of genetic polymorphisms in explaining inter-individual differences in cognitive MDMA effects, individuals with a CYP2D6 ultrarapid metabolizer genotype performed worse on semantic fluency tasks compared to a control group [43]. Specifically, those with such a genotype generated significantly fewer words within a set time frame. This study demonstrated an influence of the CYP2D6 ultrarapid metabolizer genotype on MDMA toxicity, indicating that increased enzymatic activity could lead to higher concentrations of neurotoxic metabolites, resulting in cognitive impairments. This result emphasizes the importance of considering genetic factors when assessing the cognitive effects of MDMA use. Of note, here again, specific rsID numbers for CYP2D6 polymorphisms were not mentioned in the study [43]. Overall, these results seem to support the hypothesis that CYP2D6 polymorphisms may modulate MDMA-induced effects. However, further research with larger sample sizes is needed to investigate this question more thoroughly. Indeed, as specified by Papaseit et al., the frequencies of poor metabolizers (7–10%) and ultrarapid metabolizers (less than 5%) in the Caucasian population and approximately 1.4% and 4.5% in the African-American population results in their reduced representation in MDMA clinical trials [45].

Regarding the other CYPs involved in MDMA metabolism (namely CYP2B6, CYP2C19, CYP3A4, and CYP1A2), the conversion of MDMA to MDA was negatively associated with genotypes known to convey lower CYP2C19 (rs4244285 and rs28399504 single nucleotide polymorphisms [SNP]s) or CYP2B6 (rs3745274 SNP) activities in the study conducted by Vizeli et al. [46]. Moreover, poor CYP2C19 metabolizers (rs4244285 and rs28399504 SNPs) exhibited greater cardiovascular responses to MDMA compared with other CYP2C19 genotypes [46]. In addition, regarding CYP1A2, tobacco smokers with the A/A genotype for the rs762551 SNP exhibited higher MDA blood concentrations compared to all nonsmokers with the A/C and C/C genotypes for the same SNP [46]. At any rate, once again, further research is also needed in the cases of these CYPs.

The impact of genetic COMT polymorphisms among MDMA users has also been well investigated. COMT is involved in the phase II metabolism of MDMA and in the metabolic inactivation of endogenous catecholamines such as NE or DA [4]. The COMT gene displays a major functional polymorphism at codon 158, producing a valine (val) to methionine (met) substitution (Val158Met; rs4680 SNP), resulting in three genotypes: val/val, val/met, and met/met. Carriers of the met allele exhibit lower enzyme activity compared with those homozygous for the val allele [4]. MDMA neurotoxicity and/or hepatotoxicity have been associated with COMT activity level [47,48]. Indeed, according to Perfetti et al., low COMT activity increases the concentration of HHMA, potentially leading to the increased formation of orthoquinones with subsequent consequences in terms of cell damage [48]. COMT activity level has also been associated with MDMA cognitive and cardiovascular effects. Indeed, in the study conducted by Cuyas et al., MDMA users (*n* = 60) carrying COMT val/val genotype exhibited poorer performance than paired controls on visuospatial attention and/or memory [43]. Furthermore, in another study conducted by Pardo-Lozano et al. (*n* = 27), individuals carrying COMT Val158Met polymorphism experienced increased cardiovascular effects (especially increased heart rate or even tachycardia) than others [39]. Additionally, COMT activity level has been associated with biological changes among MDMA users. Indeed, as for low CYP2D6 activity genotypes, low COMT activity genotypes have been associated with an increased risk of hyponatremia and increased production of cortisol [42,44]. Otherwise, it is also important to note that a specific polymorphism in COMT (rs165599) seems to modulate MDMA-induced negative effects on verbal fluency among MDMA users [49]. This polymorphism, which involves an A allele being replaced by a G allele, has been the subject of extensive research [50,51]. For example, a study conducted by Lamb et al. highlighted that the G allele is associated with a 24% lower messenger ribonucleic acid (mRNA) expression compared to the A allele [51]. However, the functional consequences of the rs165599 SNP in terms of COMT protein expression and/or enzymatic activity still remain not fully understood [50,51]. Such a lack of clarity underscores the complexity of linking specific genetic polymorphisms to phenotypic outcomes and highlights the necessity for further studies. The influence of metabolic enzyme polymorphisms on MDMA’s effects is summarized in the Table 1.

## 3. Genetic Factors Influencing Pharmacological MDMA Targets

Most studies on genetic variants affecting the PD of MDMA have focused on the 5HT system. 5HT is a neurotransmitter that regulates various activities in humans including behavior, mood, memory, and gastrointestinal homeostasis [52]. 5HT is synthesized in the raphe nuclei of the brain and the enterochromaffin cells of the intestinal mucosa [52]. At a molecular level, synthesis begins with L-tryptophan (L-Trp), which undergoes hydroxylation to 5-hydroxy-L-triptophan and decarboxylation to 5HT. The hydroxylation reaction requires TPH (which is considered the rate limiting enzyme of 5HT production), while the decarboxylation reaction requires aromatic L-amino acid decarboxylase. 5HT activity is regulated by its rate of synthesis, release, and metabolism. 5HT that is recycled back via the 5HT transporter (5HTT) may be stored into vesicles or metabolized by MAO [52]. The 5HT system is a primary target for many medications but also several recreational drugs like MDMA. Indeed, as described above, this compound acts as a releaser and reuptake inhibitor of 5HT. In addition, MDMA acts as a 5HTR2A weak agonist and inhibits both MAO and TPH [2,3] (Figure 1).

Several studies have pointed out associations between MDMA-induced effects and 5HTT expression [43]. 5HTT is encoded by the gene SLC6A4 (located on the human chromosome 17). A variation in the promoter region of this gene (known as serotonin-linked promoter region or 5HTTLPR) influences its transcriptional activity and regulates 5HTT expression [53]. Furthermore, another genetic variation corresponding to a variable number tandem repeat (VNTR) polymorphism in the intron 2 (5HTTVNTR) may also have functional consequences in terms of 5HTT expression [54]. In the study conducted by Roiser et al. (*n* = 56), MDMA users carrying the 5HTTLPR s/s (low functionality) genotype were the group that scored highest on the Beck Depression Inventory and performed worst in the Go/No-Go affective test, suggesting that the possession of the s allele confers particular vulnerability to disturbances in emotional processing after MDMA use [55]. It is important to note that the 5HTTLPR genotype did not affect outcomes at baseline (i.e., before MDMA use) in this study. There were no significant differences in behavioral measures or genotype frequencies between the comparison groups (cannabis users and healthy volunteers) when analyzed. This result suggests that the 5HTTLPR genotype did not influence initial outcomes prior to MDMA use [55]. Such an association between 5HTTLPR polymorphisms and mood disorders among MDMA users has also been highlighted in other studies [56,57]. Martin-Santos et al. showed a significant association between the 5HTTLPR genotype and lifetime prevalence of primary mood disorders in MDMA users (*n* = 37). Individuals carrying the 5HTTLPR s/s genotype had a higher prevalence of primary mood disorders (33.3%) compared to those with l/l or l/s genotypes (4%) [56]. In their study, Kuypers et al. observed that MDMA significantly reduced self-rated depressive feelings specifically in females carrying the 5HTTLPR s/s genotype (*n* = 63). This reduction in depressive feelings was not evident in males or in females carrying the s allele [57]. Moreover, in the study conducted by Fagundo et al. (*n* = 30), MDMA users carrying the 5HTTLPR s/s genotype performed significantly worse than the others on a verbal fluency assessment [49]. In another study, MDMA users carrying the 5HTTLPR s/s genotype exhibited poorer performance than paired controls on visuospatial attention and/or memory after MDMA consumption [43]. Otherwise, in the study conducted by Pardo-Lozano et al. (*n* = 27), subjects carrying the 5HTTLPR l/* (high functionality) genotype experienced increased cardiovascular effects (especially increased heart rate or even tachycardia) than others, and the subjects carrying 5HTTLPR s/s genotype exhibited more negative subjective effects (including dizziness, anxiety, and sedation) than others [39]. These results appear to link MDMA-induced effects to 5HTTLPR polymorphisms [58]. These data are summarized in Table 2. Furthermore, it is important to note that such an association has not been demonstrated with HTTVNTR polymorphisms. Similarly, MDMA-induced effects do not seem to depend on genetic variations within the genes encoding for TPH1 (rs1800532 and rs1799913), TPH2 (rs7305115), 5HTR1A (rs6295), 5HTR1B (rs6296), and 5HTR2A (rs6313). This may seem surprising, given the fact that this gene is highly polymorphic and that several polymorphisms have been linked to various neuropsychiatric disorders [59,60,61,62,63,64,65,66]. Otherwise, it is important to note that no study has investigated a potential link between MDMA-induced effects and genetic variations within the gene encoding for MAO.

MDMA also interacts with the DA system. Indeed, MDMA is a potent inhibitor of reuptake and a stimulator of DA release [2,3,4,5]. In addition, MDMA has antagonistic effects on DR and, more specifically, DRD2 [4,5]. DA is an endogenous catecholamine, which exerts widespread effects in humans. Within the central nervous system, DA binds to specific membrane receptors and plays a key role in the control of locomotion, learning, memory, cognition, and emotion [66]. The DA system is involved in various neurological and psychiatric disorders such as Parkinson’s disease or schizophrenia and in drug addiction [66]. DA synthesis involves several enzymes and cofactors, but the rate-limiting enzyme appears to be tyrosine hydroxylase. Furthermore, the DA system also includes the DA transporter (DAT1) and several DRs (namely D1, D2, D3, D4, and D5) with various structures and functions [66,67,68]. DAT1 is encoded by the gene SLC6A3 (located on the human chromosome 5). The most commonly investigated genetic variation within this gene is the 3’-UTR VNTR polymorphism, which regulates DAT1 expression. A variation in the number of repeats has been associated with several conditions including attention-deficit hyperactivity disorder, alcohol use disorder, and Parkinson’s disease. DRs are encoded by different genes located in different chromosomes. The gene encoding for DRD2 is located on the human chromosome 11 and appears highly polymorphic [69,70]. To the authors’ knowledge, only one study has explored the influence of variants within genes encoded for the DA system on the effects of MDMA in humans [24]. This randomized, placebo-controlled, crossover study was conducted by Vizeli et al. and investigated several polymorphisms (SNP and VNTR) within genes encoded for key players in the DA system including DAT1 (rs28363170, rs3836790, rs6347, rs11133767, rs11564774, rs460000, and rs463379 SNPs), DRD2/ANKK1 (rs1800497 SNP), DRD2 (rs6277 and rs107959 SNPs), and DRD4 (rs1805186) [24]. However, with the limit of the number of included patients (*n* = 149), none of the tested genetic polymorphisms were associated with MDMA-induced effects, suggesting that genetic variations within genes encoded for the DA system are unlikely to explain interindividual differences in the effects of MDMA in humans [24]. Importantly, the influence of variants within other genes (namely those encoded for brain-derived neurotrophic factor and the glutamate receptor subunit epsilon-2) has been explored, but no association has been demonstrated [43].

To date, only a few other PD targets of MDMA have been investigated. Bershad et al. have examined the influence of an SNP within the oxytocin receptor (OXTR) gene (rs53576 SNP) on responses to MDMA. In their study, the authors found that a high dose of MDMA (1.5 mg/kg) did not increase sociability in individuals carrying the rs35376 A/A genotype as it did in G allele carriers. Interestingly, the genotypic groups did not differ in response at the lower MDMA dose nor in cardiovascular or other subjective responses [71]. Similarly, Vizeli et al. investigated the impact of OXTR gene variations on the socioemotional effects of MDMA in humans. The study pooled data from eight double-blind, placebo-controlled studies involving 132 healthy subjects. The analysis focused on three SNPs of the OXTR gene (rs53576, rs1042778 and rs2254298 SNPs). The authors found that MDMA produced significantly greater feelings of trust in individuals with the rs1042778 TT genotypes compared with G allele carriers. They concluded that OXTR gene variations might influence certain prosocial effects of MDMA in humans, but this interpretation should be cautious due to the small sample size [72]. Otherwise, Vizeli et al. are the only authors known to have investigated the impact of genotypes within the NE system on MDMA’s effects. This may appear as a significant gap. Indeed, the NE system may play an unexpected role in the actions of MDMA and contributes to its cardiac and psychostimulant effects [73,74]. In this system, the NE transporter (NET) is a crucial player. NET is encoded by the gene SLC6A2, which is located on the human chromosome 16 (locus 16q12.2). This gene is highly polymorphic, and several SNPs have been described [75]. In their study, Vizeli et al. investigated whether the SLC6A2 rs168924, rs47958, rs1861647, rs2242446, and rs36029 SNPs influence MDMA’s cardiovascular and subjective stimulant effects. The study pooled data from eight double-blind, placebo-controlled studies involving 124 healthy subjects. The authors found that three SLC6A2 SNPs (rs1861647, rs2242446, and rs36029 SNPs) seemed to influence the cardiovascular response to MDMA, but the effect sizes were small and did not allow definitive conclusions [74]. Consequently, large-scale, controlled pharmacogenomics studies are needed to investigate the role of the NE system in MDMA’s actions. The influence of pharmacodynamics (PD) target polymorphisms on MDMA’s effects is summarized in the Table 3.

## 4. Conclusions

A certain interest has been focused on the pharmacogenomics of MDMA over the last decades. Indeed, the influence of variants within genes encoded for several enzymes involved in MDMA metabolism (especially CYP2D6 and COMT) and/or PD targets (especially the 5HT system) on the effects of MDMA in humans have been investigated. Thereby, some studies have pointed out associations between MDMA-induced effects and polymorphisms. COMT Val158Met polymorphism has, for example, been associated with cognitive and cardiovascular effects. Similarly, the 5HTTLPR s/s genotype has been associated with several MDMA-induced adverse effects, especially mood disorders. However, despite interesting results, only a few associations have been highlighted. Furthermore, some genes, in particular those encoded for MDMA targets, have been only poorly investigated, which appears as a significant gap. This reinforces the need for large-scale, controlled pharmacogenomics studies focused on a large panel of genes involved in MDMA PK and PD. Such an approach appears necessary today due to the re-emergence of psychedelic agents in Western medicine as a promising treatment for several mental conditions including PTSD or major depression disorder.

## Figures and Tables

**Table 1:** The influence of metabolic enzyme polymorphisms on MDMA’s effects.

Gene	Polymorphisms (rsId)	Minor Allele Frequency (dbSNP)	Enzyme Activity	Studies	Sample Size	Results/Outcomes	References
CYP2B6	rs3745274	T = 0.255351/35,719 (ALFA)	Low activity	Vizeli et al. (2017)	142	Altered metabolism of MDMA	[46]
CYP2C19	rs4244285	A = 0.149748/38,985 (ALFA)				Altered metabolism of MDMA Enhanced cardiovascular response	
	rs28399504	G = 0.00319/863 (ALFA)					
CYP1A2	rs762551	C = 0.318648/21,670 (ALFA)	High activity			Altered metabolism of MDMA	
CYP2D6	Not reported	N/A	Low activity	Wolff et al. (2012)	48	Increased production of cortisol	[44]
				Aitchison et al. (2012)	48	Increased risk of hyponatremia	[42]
				De la Torre (2005)	10	Altered metabolism of MDMA	[40]
				Schmid et al. (2016)	139	Increased blood pressure Altered metabolism of MDMA	[41]
			High activity	Cuyas et al. (2011)	60	Altered metabolism of MDMA Altered cognitive effects	[43]
COMT	rs4680	A = 0.489085/138,095 (ALFA)	Low activity (met/*)	Cuyas et al. (2011)	60	Altered metabolism of MDMA Altered cognitive effects	[43]
				Wolff et al. (2012)	48	Increased production of cortisol	[44]
				Aitchison et al. (2012)	48	Increased risk of hyponatremia	[42]
				Pardo-Lozano et al. (2012)	27	Increased cardiovascular effects	[39]
	rs165599	G = 0.335031/76,442 (ALFA)	Low activity?	Fagundo et al. (2010)	30	Impaired language performances	[49]

**Table 2:** The influence of 5HTTLPR polymorphisms on MDMA’s effects.

Gene	Genotypes	Studies	Sample Sizes	Results/Outcomes	References
5HTTLPR	s/s	Roiser et al. (2005)	66	Emotional disturbances	[55]
		Martin-Santos et al. (2009)	37	Mood disorders (comorbid primary mood disorder)	[56]
		Fagundo et al. (2010)	30	Impaired cognitive performance (verbal fluency)	[49]
		Cuyas et al. (2011)	60	Impaired cognitive performance (visuospatial attention and memory)	[43]
		Pardo-Lozano et al. (2012)	27	Emotional disturbances	[39]
	l/l	Kuypers et al. (2018)	63	Reduction in self-rated depressive feelings	[57]
	l/*	Pardo-Lozano et al. (2012)	27	Cardiovascular effects	[39]

**Table 3:** The influence of pharmacodynamics (PD) target polymorphisms on MDMA’s effects.

Genes	Polymorphisms (rsId)	Minor Allele Frequency (dbSNP)	Studies	Sample Size	Results/Outcomes	References
TPH1	rs1800532	T = 0.364661/23,040 (ALFA)	Vizeli et al. (2019)	125	No significant impact	[25]
	rs1799913	T = 0.369055/34,238 (ALFA)				
TPH2	rs7305115	T = 0./0 (ALFA)				
5HTR1A	rs6295	G = 0.479109/8646 (ALFA)				
5HTRIB	rs6296	G = 0.264684/13,303 (ALFA)				
5HTR2B	rs6313	A = 0.420112/145,567 (ALFA)				
DAT1	rs28363170	Not reported	Vizeli et al. (2019)	149		[24]
	rs3836790					
	rs6347	C = 0.272059/35,631 (ALFA)				
	rs11133767	T = 0.324161/26,010 (ALFA)				
	rs11564774	G = 0.230439/4353 (ALFA)				
	rs460000	C = 0./0 (ALFA)				
	rs463379	C = 0.151071/2073 (ALFA)				
DRD2/ANKK1	rs1800497	A = 0.204778/41,712 (ALFA)				
DRD2	rs6277	A = 0.48466/33,521 (ALFA)				
	rs107959	Not reported				
DRD4	rs1805186	Not reported				
OXTR	rs53576	A = 0.326459/24,474 (ALFA)	Bershad et al. (2016)	68		[72]
			Vizeli et al. (2018)	123		[73]
	rs1042778	C = 0./0 (ALFA)			Greater feelings of trust	
	rs2254298	A = 0.139783/6522 (ALFA)			No significant impact	
SLC6A2	rs168924	G = 0.147036/22,375 (ALFA)	Vizeli et al. (2018)	124		[75]
	rs47958	A = 0.433445/15,578 (ALFA)				
	rs1861647	A = 0.205928/2668 (ALFA)			Increased cardiovascular response	
	rs2242446	C = 0.283916/83,373 (ALFA)				
	rs36029	G = 0.381354/8267 (ALFA)				
